# Just to Please His Mother

**Published:** 1861-01

**Authors:** F. A. Willett

**Affiliations:** Chicago


### "JUST TO PLEASE HIS MOTHER."

BY F. A. WILLETT.

On the 9th of September, J—— A——, a boy aged about 11 years, in his play fell and tore out the left superior central incisor. He ran into the house and told his mother what had happened. Upon asking him for the tooth, he said "it was out doors, but did not know where." By diligent search it was found in the mud. After washing the mud and dirt from it, she gave it to her husband, requesting him to take the boy to Dr. Allport and have the tooth reset, as "she believed it would grow in again." Being thus urged, he put the tooth in his pocket, and brought the boy to Dr. A.'s office, about an hour after the circumstance as related above had happened. He had "no idea that it would do any good, but *to please his mother*, had brought him up to have Dr. Allport do what he could for it." An examination was made by Dr. A., and it was found that a portion of the alveolus and gum, to the extent of three lines in width by six in length, was torn up. Having no large amount of faith as to the final success of the operation, he washed the tooth of the dust, dirt and tobacco that stuck to it from the pocket, and placed it in its former bed. After pressing it up firmly, it was secured by means of silk ligatures, connecting with the adjoining teeth. The loosened portion of the alveolus and gum, which was hanging, was also pressed back into place and kept there by the lip. The boy called in every few days to let the doctor see how he was "getting along." At the end of two weeks, the ligatures were removed, as the parts had become firm enough to retain the tooth without danger of displacement. Nothing more was heard of him till a few weeks since, when he called to have it examined, as it was "getting pretty sore;" and sure enough, on examination, a large alveolar abscess was found to have formed and was about to discharge under the lip. An opening was made into the nerve cavity from the lingual surface of the tooth, the nerve extracted, and the abscess treated through this opening.

After being thoroughly treated for ten or twelve days, the nerve cavity was filled, and the case dismissed.

The tooth, at the present time, presents an appearance as healthy, and is set in the jaw nearly as firm as any of its neighbors.

*Chicago*, November 20, 1860.

